# Personality Trait Patterns Moderate the Simple Model of Environmental Citizenship

**DOI:** 10.3390/bs13020159

**Published:** 2023-02-12

**Authors:** Mykolas Simas Poškus

**Affiliations:** Department of Environmental Sciences, Vytautas Magnus University, Kristijono Donelaičio Str. 58, 44248 Kaunas, Lithuania; mykolas_poskus@mruni.eu

**Keywords:** environmental citizenship, knowledge, personality, civic participation, education

## Abstract

The current study investigates the Simple Model of Environmental Citizenship (SMEC) in a representative sample of Lithuanian emerging adults. The SMEC is a practical model of assessing environmental citizenship and is intended to be simple to use in interventions and longitudinal research. A total of 700 individuals (50% female) with a mean age of 30.6 years participated in the survey. The participants filled in a questionnaire comprising measures assessing all the components of the SMEC as well as a personality trait measure. Participants were clustered by their personality traits and the resulting profiles were used as a moderator for the SMEC. The results revealed that the SMEC functions differently for individuals possessing different personality trait patterns and that in order to promote environmental citizenship or to engage in education for environmental citizenship, different strategies might be more effective for different individuals.

## 1. Introduction

Tackling environmental issues, whether local or global, is not an easy task. While some issues might be solvable with top-down initiatives, certain problems, especially related to individual behavior, need to be tackled by communities themselves [1,2]. Some pro-environmental behaviors might be fairly common or even default in some cases (e.g., recycling) [3], while others require specific knowledge and willingness to engage in them (e.g., changing transportation habits to more sustainable options) [4,5,6]. One way of making sure that communities are empowered to make a positive contribution toward sustainability goals is to educate environmental citizens who can act as agents of change, both locally and globally [7,8].

It must be noted that environmental issues are often politicized [9,10] and presented as moral issues [11], which might lead to more impulsive behavior and the in-group biases of both individuals who view themselves as “environmentalists” and those who actively distance themselves from this group. Such biases promote in-group beliefs regardless of their factual accuracy (e.g., many environmentalists believe that nuclear energy is dangerous and produces a lot of pollution). Environmental citizens, on the other hand, are armed with the necessary factual knowledge to make accurate decisions both in their activities as agents of change and in their personal lives [12].

Currently, there is an increase in research interest on environmental citizenship and education for environmental citizenship [13,14], yet the roots of the concept are comparatively old [15]. Environmental citizenship is defined as “the active participation of citizens, in the private and public sphere, through individual and collective actions, toward solving current environmental problems and preventing the creation of new ones, parallel to developing a healthy relationship with nature” [16]. The concept does not represent one particular behavior or even a set of behaviors, but is an interactive system of pro-environmental attitudes, the willingness to learn and act, the possession of the necessary knowledge and the ability to apply it. Thus, older models of environmental citizenship tended to be impractically complicated, while current models tend to only suggest the components of environmental citizenship and have just recently begun to propose the relationships among its parts [17]. The increased interest in the topic along with the lack of a simple model to assess environmental citizenship leaves a big gap in the literature.

To address the aforementioned gap in the literature, the Simple Model of Environmental Citizenship (SMEC) was developed [17]. The model is a simplified version of the model proposed by Hawthorne and Alabaster [15]. The SMEC comprises relatively few components, all strongly grounded in the literature relevant to environmental citizenship and education for environmental citizenship, making the model suitable for both investigating environmental citizenship as a system as well as using the model for interventions, since the model mainly consists of variables (e.g., environmental knowledge, environmental education, environmental literacy) that can be directly targeted through interventions that focus on education for environmental citizenship—another area that currently is receiving increased attention from researchers [7].

**Figure 1:**
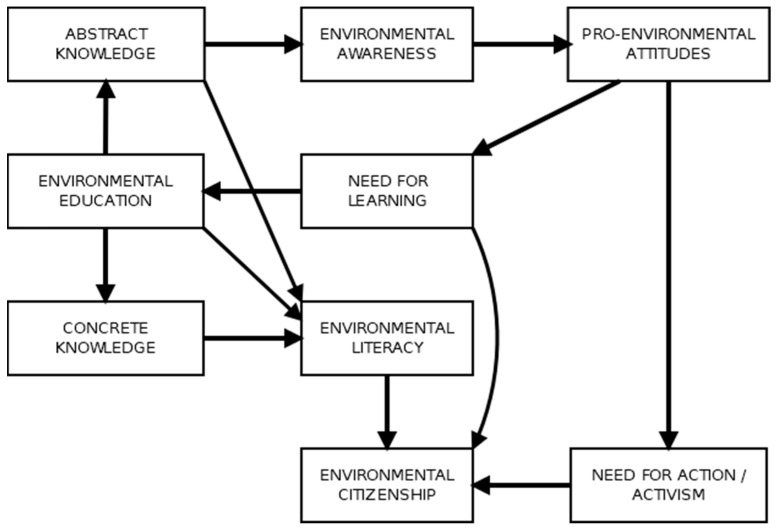
The Simple Model of Environmental Citizenship.

The current iteration of the SMEC is presented in Figure 1. While the paths specified in the figure seem to be quite robust and are supported by the previous literature, as well as by the empirical testing of the model, the model cannot be assumed to be complete and may function differently with different samples or age groups. Hence, it is prudent to adopt an exploratory approach regarding this model until a sufficient literature basis is accumulated to solidify its structure.

The current study explores the SMEC in a sample of emerging adults. In the present study, we do not assume that the model structure is final and adopt an exploratory approach toward it. Emerging adults have been chosen for sampling because of their relative ability and willingness to engage in environmental citizenship behaviors as they are young enough to be open to novel issues but mature enough to have at least some resources and capacity to participate as environmental citizens [17].

The current study adopts a person-oriented perspective [18,19,20] and uses personality trait patterns as moderators for the model. While the variable-oriented approach is more popular and indeed the Hawthorne and Alabaster [15] model includes individual characteristics as parts of the model, the SMEC consists of constructs that are subject to change, whereas personality traits are stable, therefore they cannot be meaningfully included in such a model as additional components [21]. Additionally, linear relationships between the components of the SMEC and personality traits would carry little practical insight as they would not allow us to make any inferences about different individuals [18].

Through a person-oriented approach and by deriving clusters of individuals, we enable a more precise understanding of how environmental citizenship manifests in different groups of individuals, thus providing us with insight into its various parts for interventions and educational approaches.

## 2. Materials and Methods

### 2.1. Sample Characteristics

The sample consists of 700 emerging adults aged from 20 to 39 years, with a mean age of 30.6 years (349 males, 351 females). The sample was constructed in such a way that it representatively reflects Lithuanian emerging adults from all areas of the country based on official statistics data available in the country.

### 2.2. Procedure

A surveying company gathered the data via an online questionnaire. Data were gathered from 20 July 2022 until 7 September 2022. The questionnaire was anonymous and the respondents gave their active consent to participate in the survey.

### 2.3. Measures

This study uses only a part of the data gathered during the survey; therefore only the variables used for the present study will be described. Questions and scales were presented to the participants in the order they are described below.

#### 2.3.1. Personality Traits

The BFI2 questionnaire [22] was used was used to assess personality traits of the participants. The questionnaire consists of 60 items, with 12 items for each of the Big Five traits. The internal consistency values for extraversion (ω = 0.725), agreeableness (ω = 0.733), conscientiousness (ω = 0.768), neuroticism (ω = 0.831), and openness (ω = 0.778) were acceptable. Responses were rated on Likert scale ranging from 1 (completely unlike me) to 5 (completely like me).

#### 2.3.2. Need for Learning

The need for learning was assessed with a 10-item scale adapted from the Attitude/Motivation Test Battery [23] and were previously used in similar research [17]. The scale assesses one’s need to learn about environmental issues and whether one perceives learning about these issues as worthwhile. Responses were rated on a 5-point Likert scale from 1 (completely disagree) to 5 (completely agree). An example of an item is as follows: “If I had a chance to learn about how I can better take care of the environment, I would jump at the opportunity”. The scale showed excellent internal consistency (ω = 0.902).

#### 2.3.3. Abstract Environmental Knowledge

To assess abstract environmental knowledge, a measure used in previous research with the SMEC [17], which was adapted from Kim and Stepchenkova [24] and Mohiuddin et al. [4], was used. Abstract environmental knowledge, as opposed to factual knowledge [25], deals with perceived ability to find relevant answers to environmentally relevant questions. The scale consists of 5 items, each rated on a Likert scale from 1 (completely disagree) to 5 (completely agree). An example of an item is as follows: “I understand the various labels on products that provide information on their environmental impact”. The scale demonstrated good internal consistency (ω = 0.828).

#### 2.3.4. Concrete Environmental Knowledge

An objective knowledge test [26] comprising 26 items was used to assess the concrete environmental knowledge of the participants. We used the version of the test that was adapted for the Lithuanian context and updated to reflect the most current state of knowledge [17]. The test consisted of questions regarding various environmental issues and provided five alternative answers to each question, with one of them being the correct answer. The scores were calculated by adding up the number of correct answers to the test with a maximum possible score of 26. The internal consistency of the measure was good (KR-20 = 0.872).

#### 2.3.5. Environmental Awareness (Consciousness)

The measure for environmental awareness was adapted from Mohiuddin et al. [4] and was previously used in testing the SMEC [17]. The measure consists of four items rated on a Likert scale from 1 (completely disagree) to 5 (completely agree). An example of an item is as follows: “I know what the consequences of climate change are”. The scale demonstrated good internal consistency (ω = 0.819).

#### 2.3.6. Environmental Attitudes

The New Environmental Paradigm (NEP) scale was used to assess environmental attitudes [27,28]. The scale consists of 15 items that address one’s attitudes regarding one’s plane in nature and the balance of nature and human activity [27]. An example of an item is as follows: “when humans interfere with nature it often produces disastrous consequences”. Items were rated on a Likert scale from 1 (completely disagree) to 5 (completely agree). The internal consistency of the scale was acceptable (ω = 0.791).

#### 2.3.7. Environmental (Self-)Education

Environmental (self-)education was assessed with 5 items directed at one’s engagement with educational materials regarding environmental issues in the past month. The scale was used in previous research testing the SMEC [17]. Items were rated on a Likert scale ranging from 1 (completely disagree) to 5 (completely agree). An example of an item is as follows: “During the past month I researched environmental questions at least a couple of times”. The scale demonstrated excellent internal consistency (ω = 0.902).

#### 2.3.8. Environmental Literacy

Environmental literacy was assessed with a measure developed by Hadjichambis and Paraskeva-Hadjichambi [29]. The measure consists of 11 items rated on a Likert scale from 1 (completely unaware) to 5 (completely aware). An example of an item is as follows: “How to contribute to the prevention of environmental problems”. The scale demonstrated excellent internal consistency (ω = 0.919).

#### 2.3.9. Environmental Citizenship

Environmental citizenship was assessed with a 3-item scale developed by Hadjichambis and Paraskeva-Hadjichambi [29]. The items were rated on a Likert scale ranging from 1 (completely disagree) to 5 (completely agree). An example of an item is as follows: “I would try to change society in such a way that it becomes more environmentally friendly”. The scale demonstrated good internal consistency (ω = 0.827).

#### 2.3.10. Need for Action

The need for action was assessed with a 6-item scale developed by Hadjichambis and Paraskeva-Hadjichambi [29]. Items were rated on a 4-point scale (1—I have done that in the past half year, 2—I have done that in the past year, 3—I have done that but more than a year ago, 4—I have never done that). An example of an item is as follows: “Have you participated in an environmental action group?” The scale demonstrated good internal consistency (ω = 0.887).

### 2.4. Analysis Strategy and Data Availability

In the present study, both person-oriented and variable-oriented approaches are used [20,30,31]. First, a linear model of SMEC is computed, then the participants of the study are clustered into groups of similar personality types, then a linear multigroup structural equation model is run to see if personality trait patterns moderate the SMEC. We adopt an exploratory approach, allowing the data to shape the model, as the SMEC is still being developed.

The data used in the present study (https://osf.io/sve87), as well as the pre-registration of the study (https://osf.io/czf68), are publicly available on the OSF.

## 3. Results

The descriptive statistics of all the variables used in further analyses are presented in Table 1. Based on the skewness and kurtosis values, we assume that the data are suitable for use in linear models. Since the SMEC [17] has not yet been tested in a representative sample of Lithuanian emerging adults, we proceeded with structural equation modelling, allowing for data-driven suggestions for potential paths for solutions that would both make theoretical sense and would improve the model’s fit to the data.

Several previously unspecified paths were added to the SMEC, namely: paths leading from pro-environmental attitudes, environmental literacy, and activism toward concrete environmental knowledge; a path leading from the need for learning toward abstract knowledge; and paths from environmental (self-)education and environmental awareness toward the need for action/activism. The model (Table 2) fit the data reasonably well and can be used for further exploration intended to determine whether the model functions the same for different individuals.

**Figure 2:**
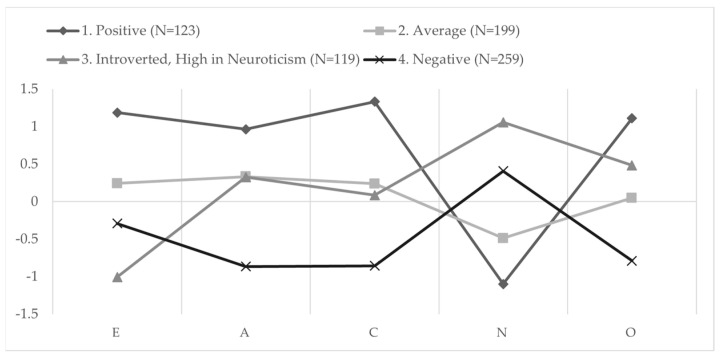
Cluster profiles based on the Big Five personality traits.

To continue with the intended person-oriented analysis of the model, a K-means cluster analysis using the Hartigan–Wong algorithm was used to find a fixed four-factor solution for the Big Five personality traits (Figure 2). Four factors were chosen based on previous research in Lithuanian samples [21,32,33] and yielded similar results, with the positive and negative clusters being completely reproduced and some variation shown in the remaining two clusters. Since previous similar research in Lithuania was conducted with samples of adolescents, it is expected to find some differences in personality trait patterns, as one’s personality, although considered as relatively stable throughout one’s lifespan [34,35,36,37], is still developing in adolescence [38], whereas this sample consists of emerging adults.

The four clusters differ qualitatively, with the Positive cluster grouping individuals with traits that are usually considered more socially desirable and the Negative cluster grouping individuals with traits that are usually regarded as less socially desirable [39,40]. The Average cluster is characterized by having mostly average expressions regarding all traits and tending to be slightly more emotionally stable than the average. The ‘Introverted, High in Neuroticism’ cluster consists of individuals that tend to keep to themselves and are less emotionally stable as well as more cautious. It needs to be emphasized that all personality trait patterns have adaptive value and simply result in different strategies that individuals take toward achieving their goals [37,41,42]. A further analysis (see Appendix A, Table A1) indicated that the sexes are not equally represented in each cluster, with cluster 3 being more common for females and cluster 4 being more common for males. Such differences, however, are expected since there are robust evidence of personality differences between the sexes and the clustering solution is consistent with the literature on these differences [43,44].

Prior to conducting a multigroup SEM analysis, a series of ANOVAs were run to see whether the identified clusters differ significantly regarding the variables of the model. In all cases, group differences were found, thus solidifying the choice to conduct a multigroup analysis of the SMEC (see Appendix A for ANOVAs and descriptive statistics, Table A2 and Table A3).

The coefficients of the multigroup analysis are presented in Table 3. Overall, the model demonstrated reasonable fit, although the observed model fit indices indicate that the model still needs improvement in future research.

Overall, the analysis supports the assumption that personality trait patterns moderate environmental citizenship (see Appendix A for means). To summarize, the path leading from environmental literacy toward environmental citizenship is the strongest for cluster 4, even though this cluster has the lowest environmental literacy scores. For the same cluster, the path from environmental (self-)education toward environmental literacy is the strongest when compared to other clusters, and the fourth cluster has comparably high scores of self-reported environmental (self-)education, with only the first cluster having higher scores. For the fourth cluster, environmental (self-)education has the strongest relationship with concrete environmental knowledge, although the Betas are comparable for all clusters. What needs to be noted here is that environmental (self-)education leads to lower objective knowledge scores for all clusters. Although cluster 4 has the lowest scores in abstract environmental knowledge, the path from abstract knowledge toward environmental awareness is strongest in this cluster. Both cluster 4 and cluster 1 have comparatively high Betas leading from environmental awareness toward pro-environmental attitudes; however, cluster 4 has the lowest awareness scores while cluster 1 has the highest. The path leading from pro-environmental attitudes toward the need for learning is significant only for cluster 4 and the effect is negative, while an insignificant effect of similar magnitude is positive for cluster 1. The clusters differ in their pro-environmental attitudes as well, with cluster 4 having the lowest scores, while cluster 1 has comparatively high scores. The path leading from the need for action/activism toward concrete knowledge is only significant for the fourth and the second clusters, but cluster 4 is the lowest in scores of the need for action/activism, while cluster 2 has comparatively high scores. The fourth cluster has the lowest scores of the need for learning but has the highest effect for the path leading from the need for learning toward abstract knowledge. Similarly, the fourth cluster (which has the lowest scores in environmental awareness) has the only significant path among the clusters leading from environmental awareness toward the need for action/activism.

The third cluster, similarly to the second cluster, has a significant path leading from concrete environmental knowledge toward environmental literacy, yet the effect is negative. Similarly, the third cluster is similar to the first cluster in the path leading from pro-environmental attitudes toward concrete knowledge, with both clusters having comparatively higher effects. The second cluster has the highest effect size among the clusters for the path leading from abstract environmental knowledge toward environmental literacy, although the first, second, and third clusters have comparatively similar scores for abstract knowledge. The path leading from environmental awareness toward concrete knowledge is significant for both the second and the fourth clusters, yet the fourth cluster is the lowest in environmental awareness scores. The first cluster has the highest effect leading from the need for learning toward environmental citizenship and among all of the clusters it has the highest scores for the need for learning. A similar pattern is seen for the path leading from the need for learning toward environmental (self-)education in which the first cluster has the strongest effect for the path. The path leading from environmental (self-)education toward abstract knowledge is only significant for the first cluster and the effect is size is medium. While the path leading from environmental (self-)education toward the need for action/activism is negative for all clusters, the highest effect size is observed in the first cluster. While the model in all clusters can explain a comparatively similar amount of variance in environmental citizenship, cluster 4 has the highest R^2^ value, while cluster 2 has the lowest (Table 4).

## 4. Discussion

Person-oriented research that uses personality trait clusters as grouping variables has previously investigated pro-environmental behaviors [21,33] and such approaches show great promise in uncovering not only how different traits relate to various outcomes, but how different individuals, possessing certain patterns of traits, act. In the present study, we managed to mostly replicate previous findings in terms of personality traits patterns found in Lithuanian samples [21,33] and have uncovered differences in the functioning of the SMEC in these clusters of individuals.

**Figure 3:**
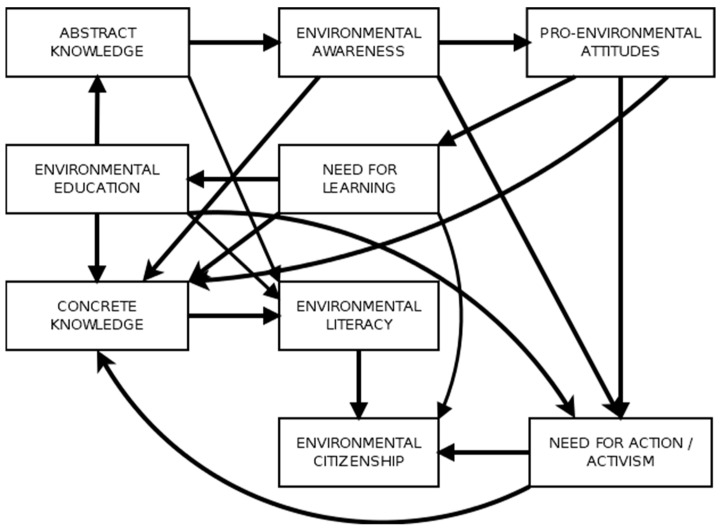
The updated Simple Model of Environmental Citizenship.

The SMEC, while not yet a finished and robust model, shows promise but also needs more work in order to simplify it further so as to reduce the number of paths and their complexity. It must be noted that the SMEC is a model developed with education for environmental citizenship in mind and that other models might be more appropriate for the study of pro-environmental behavior in general. The main drawback of the SMEC is that it is based on fairly old research and future studies should adopt an exploratory SEM approach to the model to see whether the proposed paths in the model are indeed robust. The current functioning version of the model is depicted in Figure 3.

In the present study, the Negative cluster, characterized by traits that are usually perceived as the least socially desirable [39,40], emerged as the least informed regarding environmental issues and the least engaged in environmental citizenship behavior overall. Although individuals in this cluster perceive that they engage in learning about environmental issues just the same as individuals in the other clusters, they have insufficient concrete knowledge and the desire to learn about environmental issues; they also tend to have the least positive attitudes toward the environment. This is consistent with previous research that indicated that individuals possessing this trait pattern are the least willing to engage in pro-environmental behaviors [33] and it takes more effort to persuade them to adopt pro-environmental behaviors [21]. On the model level, the data suggest that it might be best to target environmental literacy and environmental knowledge if we are to encourage these individuals to engage in informed environmental action.

The Positive cluster, characterized by traits that are usually regarded as more socially desirable, presents the opposite picture. This cluster groups individuals that have the best factual knowledge regarding environmental issues, although neither this cluster, nor any of the other clusters reached the maximum score in the knowledge test. This cluster also has the most positive pro-environmental attitudes and is more engaged in environmental citizenship behaviors, although not in environmental activism. This might be an effective area to explore in the future as informed and solution-oriented activism is gravely needed in fighting environmental issues [17]. The individuals in this cluster tend to have higher scores of subjectively perceived abstract environmental knowledge, but also show the highest motivation to further learn about environmental issues. As a matter of fact, we observe the highest effects leading from the need for learning toward both environmental citizenship and environmental (self-)education, demonstrating that quality education materials have the potential to further encourage individuals of this cluster to engage in environmental citizenship behavior. Again, this is consistent with the previous literature that used clustering approaches [21,33].

The Average cluster, characterized by mostly average expressions of personality traits, as well as the Introverted, High in Neuroticism cluster tend to be similar in how the SMEC functions for these clusters. Often having mean scores of the assessed variables that are in between the Positive and Negative clusters, the Average and Introverted, High in Neuroticism clusters can be best encouraged to engage in environmental citizenship behaviors by targeting their environmental literacy, need for learning, and abstract environmental knowledge. Of course, interventions should also include factual knowledge, but the focus likely should be on ways of acquiring quality information, not just presenting facts.

Overall, the present study sheds some light on how different individuals could be approached in promoting their environmental citizenship [21,33]. Based on the differences in how the SMEC functions among clusters, it becomes apparent that a one-size-fits-all approach might not be the best course of action when engaging students in education in environmental citizenship and that individual differences should be taken into account [45]. While educational interventions should use factual data [6,17,46], the present study shows that understanding environmental issues is not the only or even a significant predictor of environmental citizenship behavior when other variables are included in the model. This indicates that interventions should capture a variety of variables of the SMEC in order to be effective, not only for the fraction of those who receive it, but for everyone.

### 4.1. Future Directions

There are several directions future research on environmental citizenship could go. Firstly, longitudinal investigations of the SMEC could uncover whether and how environmental citizenship changes over time. Both intervention studies and longitudinal studies without any intervention would add to the understanding of both environmental citizenship and how the SMEC performs in various samples and under varying circumstances.

Future research could also look into the SMEC in other cultures to determine whether the model is culturally robust. Additionally, the model is by no means complete and is subject to change; therefore, future attempts to simplify the model, perhaps merging some components in broader constructs, would be very useful.

Lastly, the role of political orientation on environmental citizenship should also be investigated. Perhaps the most informative way to do so is to see whether political orientation moderates the various paths of the SMEC leading toward environmental citizenship. This would be a logical continuation of the person-oriented approach regarding environmental citizenship, as political beliefs are largely stable over time and, to a substantial degree, innate [47].

### 4.2. Limitations

As with all research, the present study has some limitations. The main limitation of the present study is the inability to run a full SEM analysis with all latent variables. While the sample used in this study is large and representative, after clustering individuals based on their personality traits, we are left with groups that are roughly a quarter of the initial sample and we are therefore forced to use path-analytical SEM.

The SMEC is a newly developed model, and this is the first study to investigate it in a representative sample from a single country. Future research should not be focused on replicating the paths of the model as they are depicted in the present study, but on searching for an even simpler structure of the model and for paths that work best in the particular cultural context the study is conducted in. In general, the literature must expand substantially before we can solidify the structure of the SMEC. One of the ways forward would be to use meta-SEM to assess the robustness of the model structure.

## Figures and Tables

**Table 1:** Descriptive statistics of all variables used.

Variable	M	SD	S	K	*r*												
					1	2	3	4	5	6	7	8	9	10	11	12	13
1. Abstract knowledge	3.519	0.833	−0.3759	−0.0461	—												
2. Need for learning	3.254	0.817	−0.1752	0.1687	0.487 ***	—											
3. Environmental citizenship	3.297	0.887	−0.4268	0.2147	0.438 ***	0.546 ***	—										
4. Need for action/ Activism	3.500	0.667	−1.4865	1.5956	−0.025	−0.093*	−0.118 **	—									
5. Concrete knowledge	14.554	5.926	−0.5713	−0.7540	0.106 ***	−0.030	−0.006	0.471 ***	—								
6. Environmental awareness	3.975	0.775	−0.7218	0.3737	0.380 ***	0.346 ***	0.291 ***	0.275 ***	0.439 ***	—							
7. Pro-environmental attitudes	3.508	0.560	0.1655	−0.2367	0.121 **	0.151 ***	0.105 **	0.239 ***	0.515 ***	0.527 ***	—						
8. Environmental (self-)education	2.848	1.070	−0.1737	−0.7074	0.340 ***	0.590 ***	0.490 ***	−0.310 ***	−0.297 ***	0.099 **	−0.088 *	—					
9. Environmental literacy	3.138	0.726	−0.2141	0.8693	0.506 ***	0.403 ***	0.590 ***	−0.217 ***	−0.173 ***	0.200 ***	−0.081 *	0.599 ***	—				
10. Extraversion	3.158	0.548	−0.0716	0.1334	0.300 ***	0.272 ***	0.291 ***	−0.124 **	−0.055	0.134 ***	−0.045	0.226 ***	0.316 ***	—			
11. Agreeableness	3.555	0.539	0.1092	−0.5333	0.221 ***	0.188 ***	0.137 ***	0.215 ***	0.312 ***	0.285 ***	0.286 ***	−0.034	0.059	0.228 ***	—		
12. Conscientiousness	3.598	0.570	0.1614	−0.5608	0.257 ***	0.216 ***	0.178 ***	0.173 ***	0.355 ***	0.307 ***	0.262 ***	−0.015	0.098 **	0.378 ***	0.526 ***	—	
13. Neuroticism	2.817	0.704	0.0782	−0.1259	−0.182 ***	−0.065	−0.122 **	0.027	−0.108 **	−0.062	0.028	−0.037	−0.145 ***	−0.558 ***	−0.368 ***	−0.411 ***	—
14. Openness	3.453	0.633	0.2581	−0.3228	0.302 ***	0.283 ***	0.249 ***	0.055	0.305 ***	0.301 ***	0.319 ***	0.118 **	0.231 ***	0.363 ***	0.424 ***	0.529 ***	−0.262 ***

***Notes.*** S—skewness, K—kurtosis, * *p* < 0.05, ** *p* < 0.01, *** *p* < 0.001.

**Table 2:** Results of the estimated structural model.

Dependent Variable	Predictor	Estimate	SE	95% CI		β	z	*p*
				Lower	Upper			
Environmental citizenship	Environmental literacy	0.54251	0.04677	0.45085	0.63418	0.44	11.6	<0.001
Environmental citizenship	Need for learning	0.40051	0.04406	0.31415	0.48687	0.3661	9.09	<0.001
Environmental citizenship	Need for action/Activism	0.01716	0.03934	−0.05993	0.09426	0.0127	0.436	0.663
Environmental literacy	Abstract knowledge	0.31364	0.03166	0.25159	0.37569	0.3607	9.907	<0.001
Environmental literacy	Environmental (self-)education	0.3079	0.02512	0.25867	0.35713	0.4547	12.259	<0.001
Environmental literacy	Concrete knowledge	−0.00938	0.00339	−0.01603	−0.00273	−0.075	−2.764	0.006
Concrete knowledge	Environmental (self-)education	−1.14526	0.158	−1.45494	−0.83558	−0.2115	−7.248	<0.001
Environmental (self-)education	Need for learning	0.77176	0.0401	0.69317	0.85036	0.589	19.247	<0.001
Abstract knowledge	Environmental (self-)education	0.06304	0.03552	−0.00658	0.13265	0.0809	1.775	0.076
Environmental awareness	Abstract knowledge	0.34179	0.04018	0.26303	0.42055	0.3675	8.506	<0.001
Pro-environmental attitudes	Environmental awareness	0.37507	0.02257	0.33085	0.4193	0.5194	16.621	<0.001
Need for action/Activism	Pro-environmental attitudes	0.07853	0.04846	−0.01645	0.1735	0.0665	1.621	0.105
Need for learning	Pro-environmental attitudes	0.08791	0.06684	−0.04309	0.21892	0.0602	1.315	0.188
Concrete knowledge	Pro-environmental attitudes	3.36447	0.37742	2.62475	4.1042	0.325	8.914	<0.001
Concrete knowledge	Environmental awareness	1.6657	0.26857	1.13931	2.19209	0.2228	6.202	<0.001
Concrete knowledge	Need for action/Activism	2.40751	0.30572	1.80831	3.0067	0.2748	7.875	<0.001
Concrete knowledge	Need for learning	0.43961	0.04895	0.34367	0.53555	0.4308	8.981	<0.001
Need for action/Activism	Environmental (self-)education	−0.20652	0.02148	−0.24862	−0.16441	−0.3342	−9.613	<0.001
Need for action/Activism	Environmental awareness	0.23484	0.04785	0.14105	0.32864	0.2752	4.908	<0.001

***Notes.*** MLM estimation was used, robust standard errors were computed. CI—confidence interval. CFI = 0.956, TLI = 0.907, SRMR = 0.052, RMSEA = 0.093, χ^2^(17) = 121, *p* < 0.001. R^2^_(Environmental citizenship)_ = 0.47, R^2^_(Environmental literacy)_ = 0.47, R^2^_(Concrete knowledge)_ = 0.42, R^2^_(Environmental (self-)education)_ = 0.35, R^2^_(Abstract knowledge)_ = 0.24, R^2^_(Environmental awareness)_ = 0.15, R^2^_(Pro-environmental attitudes)_ = 0.28, R^2^_(Need for action/Activism)_ = 0.18, R^2^_(Need for learning)_ = 0.02.

**Table 3:** Estimates of the multigroup structural equation, comparing the SMEC in clusters.

Cluster	Dependent variable	Predictor	Estimate	SE	95% CI		β	z	*p*
					Lower	Upper			
1	Environmental citizenship	Environmental literacy	0.33109	0.12089	0.09416	0.56803	0.2664	2.739	0.006
	Environmental citizenship	Need for learning	0.45638	0.10988	0.24103	0.67173	0.4414	4.154	<0.001
	Environmental citizenship	Need for action/Activism	−0.15311	0.13756	−0.42272	0.1165	−0.1046	−1.113	0.266
	Environmental literacy	Abstract knowledge	0.34948	0.07941	0.19385	0.50512	0.3667	4.401	<0.001
	Environmental literacy	Environmental (self-)education	0.27481	0.04799	0.18075	0.36887	0.4422	5.726	<0.001
	Environmental literacy	Concrete knowledge	−0.01301	0.01128	−0.03512	0.0091	−0.0735	−1.154	0.249
	Concrete knowledge	Environmental (self-)education	−0.71148	0.33476	−1.36761	−0.05536	−0.2027	−2.125	0.034
	Environmental (self-)education	Need for learning	0.95889	0.07498	0.81194	1.10584	0.7163	12.789	<0.001
	Abstract knowledge	Environmental (self-)education	0.18683	0.07779	0.03437	0.3393	0.2865	2.402	0.016
	Environmental awareness	Abstract knowledge	0.29965	0.10712	0.08971	0.5096	0.3283	2.797	0.005
	Pro-environmental attitudes	Environmental awareness	0.44846	0.08247	0.28682	0.61009	0.5296	5.438	<0.001
	Need for action/Activism	Pro-environmental attitudes	−0.15998	0.13161	−0.41794	0.09797	−0.1529	−1.216	0.224
	Need for learning	Pro-environmental attitudes	0.20362	0.16952	−0.12863	0.53587	0.1374	1.201	0.23
	Concrete knowledge	Pro-environmental attitudes	2.7817	0.7809	1.25116	4.31223	0.3994	3.562	<0.001
	Concrete knowledge	Environmental awareness	0.6998	0.63101	−0.53696	1.93656	0.1187	1.109	0.267
	Concrete knowledge	Need for action/Activism	0.27935	0.59557	−0.88795	1.44665	0.042	0.469	0.639
	Abstract knowledge	Need for learning	0.22965	0.12102	−0.00755	0.46685	0.2631	1.898	0.058
	Need for action/Activism	Environmental (self-)education	−0.20552	0.0472	−0.29802	−0.11302	−0.3897	−4.355	<0.001
	Need for action/Activism	Environmental awareness	0.19772	0.17663	−0.14846	0.54391	0.2231	1.119	0.263
2	Environmental citizenship	Environmental literacy	0.4052	0.09883	0.2115	0.59891	0.3261	4.1	<0.001
	Environmental citizenship	Need for learning	0.42652	0.08375	0.26238	0.59067	0.4251	5.093	<0.001
	Environmental citizenship	Need for action/Activism	−0.07438	0.07949	−0.23018	0.08143	−0.0481	−0.936	0.349
	Environmental literacy	Abstract knowledge	0.33488	0.0537	0.22963	0.44012	0.4008	6.236	<0.001
	Environmental literacy	Environmental (self-)education	0.18145	0.04568	0.09191	0.27099	0.2984	3.972	<0.001
	Environmental literacy	Concrete knowledge	−0.01713	0.00769	−0.03221	−0.00206	−0.1318	−2.227	0.026
	Concrete knowledge	Environmental (self-)education	−1.10311	0.28454	−1.66079	−0.54543	−0.2359	−3.877	<0.001
	Environmental (self-)education	Need for learning	0.73822	0.07779	0.58575	0.89069	0.5561	9.49	<0.001
	Abstract knowledge	Environmental (self-)education	0.10401	0.06436	−0.02214	0.23016	0.1429	1.616	0.106
	Environmental awareness	Abstract knowledge	0.18886	0.07143	0.04886	0.32885	0.2083	2.644	0.008
	Pro-environmental attitudes	Environmental awareness	0.32129	0.05261	0.21819	0.4244	0.4161	6.108	<0.001
	Need for action/Activism	Pro-environmental attitudes	−0.02346	0.05456	−0.1304	0.08348	−0.0245	−0.43	0.667
	Need for learning	Pro-environmental attitudes	0.17251	0.11623	−0.05528	0.40031	0.1167	1.484	0.138
	Concrete knowledge	Pro-environmental attitudes	2.36512	0.66661	1.05859	3.67166	0.2576	3.548	<0.001
	Concrete knowledge	Environmental awareness	2.03722	0.52834	1.00169	3.07275	0.2874	3.856	<0.001
	Concrete knowledge	Need for action/Activism	1.77044	0.56442	0.6642	2.87669	0.185	3.137	0.002
	Abstract knowledge	Need for learning	0.22116	0.08709	0.05047	0.39185	0.2289	2.539	0.011
	Need for action/Activism	Environmental (self-)education	−0.18423	0.03963	−0.26191	−0.10656	−0.3771	−4.649	<0.001
	Need for action/Activism	Environmental awareness	0.1248	0.07567	−0.02352	0.27311	0.1685	1.649	0.099
3	Environmental citizenship	Environmental literacy	0.60617	0.08574	0.43812	0.77422	0.5207	7.07	<0.001
	Environmental citizenship	Need for learning	0.3239	0.0822	0.16278	0.48502	0.2865	3.94	<0.001
	Environmental citizenship	Need for action/Activism	−0.06537	0.08793	−0.23772	0.10698	−0.0302	−0.743	0.457
	Environmental literacy	Abstract knowledge	0.3374	0.06978	0.20065	0.47416	0.3712	4.836	<0.001
	Environmental literacy	Environmental (self-)education	0.35215	0.05353	0.24723	0.45706	0.4866	6.579	<0.001
	Environmental literacy	Concrete knowledge	−0.02929	0.01322	−0.0552	−0.00337	−0.1439	−2.215	0.027
	Concrete knowledge	Environmental (self-)education	−0.77202	0.34301	−1.44431	−0.09972	−0.2171	−2.251	0.024
	Environmental (self-)education	Need for learning	0.80038	0.09737	0.60953	0.99123	0.5963	8.22	<0.001
	Abstract knowledge	Environmental (self-)education	0.01612	0.09239	−0.16497	0.1972	0.0202	0.174	0.862
	Environmental awareness	Abstract knowledge	0.25429	0.05953	0.13762	0.37097	0.376	4.272	<0.001
	Pro-environmental attitudes	Environmental awareness	0.29097	0.07826	0.13758	0.44436	0.3304	3.718	<0.001
	Need for action/Activism	Pro-environmental attitudes	0.0611	0.09115	−0.11755	0.23974	0.0746	0.67	0.503
	Need for learning	Pro-environmental attitudes	0.14997	0.16527	−0.17395	0.4739	0.0954	0.907	0.364
	Concrete knowledge	Pro-environmental attitudes	2.82603	0.81263	1.23331	4.41875	0.3767	3.478	<0.001
	Concrete knowledge	Environmental awareness	−0.56422	0.63731	−1.81333	0.68488	−0.0854	−0.885	0.376
	Concrete knowledge	Need for action/Activism	−0.1181	0.81516	−1.71578	1.47959	−0.0129	−0.145	0.885
	Abstract knowledge	Need for learning	0.3245	0.12147	0.08642	0.56258	0.3037	2.671	0.008
	Need for action/Activism	Environmental (self-)education	−0.09697	0.03483	−0.16523	−0.02872	−0.2495	−2.785	0.005
	Need for action/Activism	Environmental awareness	−0.06008	0.06454	−0.18657	0.06641	−0.0832	−0.931	0.352
4	Environmental citizenship	Environmental literacy	0.69509	0.06601	0.56572	0.82446	0.5699	10.531	<0.001
	Environmental citizenship	Need for learning	0.28228	0.0684	0.14822	0.41633	0.2383	4.127	<0.001
	Environmental citizenship	Need for action/Activism	0.063	0.04487	−0.02493	0.15094	0.0555	1.404	0.16
	Environmental literacy	Abstract knowledge	0.20463	0.05681	0.09328	0.31598	0.2281	3.602	<0.001
	Environmental literacy	Environmental (self-)education	0.41912	0.05189	0.31742	0.52082	0.5753	8.077	<0.001
	Environmental literacy	Concrete knowledge	−0.0045	0.00571	−0.01569	0.00668	−0.0389	−0.789	0.43
	Concrete knowledge	Environmental (self-)education	−1.78387	0.27685	−2.3265	−1.24125	−0.2835	−6.443	<0.001
	Environmental (self-)education	Need for learning	0.7676	0.07277	0.62498	0.91022	0.5757	10.549	<0.001
	Abstract knowledge	Environmental (self-)education	0.10126	0.06019	−0.0167	0.21923	0.1247	1.683	0.092
	Environmental awareness	Abstract knowledge	0.40568	0.07831	0.2522	0.55915	0.4045	5.181	<0.001
	Pro-environmental attitudes	Environmental awareness	0.29161	0.03221	0.22848	0.35474	0.5237	9.054	<0.001
	Need for action/Activism	Pro-environmental attitudes	0.2273	0.09423	0.04262	0.41199	0.1317	2.412	0.016
	Need for learning	Pro-environmental attitudes	−0.31684	0.13761	−0.58655	−0.04712	−0.1915	−2.302	0.021
	Concrete knowledge	Pro-environmental attitudes	2.83102	0.69431	1.4702	4.19184	0.204	4.077	<0.001
	Concrete knowledge	Environmental awareness	1.58359	0.44167	0.71794	2.44924	0.2049	3.585	<0.001
	Concrete knowledge	Need for action/Activism	2.84791	0.50661	1.85497	3.84085	0.3542	5.621	<0.001
	Abstract knowledge	Need for learning	0.60595	0.07941	0.4503	0.7616	0.5597	7.63	<0.001
	Need for action/Activism	Environmental (self-)education	−0.19909	0.04022	−0.27792	−0.12027	−0.2544	−4.95	<0.001
	Need for action/Activism	Environmental awareness	0.29327	0.07392	0.14839	0.43815	0.3051	3.967	<0.001

***Notes.*** Clusters are: 1—Positive; 2—Average; 3—Introverted, High in Neuroticism; 4—Negative. MLM estimation was used, and robust standard errors were computed. CI—confidence interval. CFI = 0.951, TLI = 0.896, SRMR = 0.057, RMSEA = 0.093, χ^2^(68) = 172, *p* < 0.001.

**Table 4:** R^2^ values for each predicted variable among clusters.

	Cluster			
	1	2	3	4
Environmental citizenship	0.431	0.388	0.488	0.502
Environmental literacy	0.500	0.351	0.499	0.513
Concrete knowledge	0.233	0.326	0.163	0.452
Environmental (self-)education	0.518	0.310	0.356	0.328
Abstract knowledge	0.276	0.115	0.107	0.378
Environmental awareness	0.128	0.049	0.148	0.120
Pro-environmental attitudes	0.296	0.178	0.116	0.236
Need for action/Activism	0.173	0.159	0.071	0.196
Need for learning	0.041	0.020	0.017	0.014

***Notes.*** Clusters are 1—Positive; 2—Average; 3—Introverted, High in Neuroticism; 4—Negative.

## Data Availability

Data as well as the pre-registration of the study are available on the OSF platform.

## Appendix A

**Table A1:** Frequencies of participants’ sex compared among clusters.

	Cluster				Total
	1	2	3	4	
Male	57	93	34	165	349
Female	66	106	85	94	351
Total	123	199	119	259	700

***Notes.*** Cluster are: 1—Positive; 2—Average; 3—Introverted, High in Neuroticism; 4—Negative. Χ^2^(3) = 42.8, *p* < 0.001.

**Table A2:** ANOVA results for all SMEC variables compared among clusters.

	Dependent Variable	Sum of Squares	df	Mean Square	F	*p*
Cluster differences	Concrete knowledge	5305.9	3	1768.647	63.96	<0.001
	Environmental awareness	55.9	3	18.634	35.64	<0.001
	Pro-environmental attitudes	35.3	3	11.764	44.59	<0.001
	Environmental (self-)education	17.5	3	5.827	5.18	0.002
	Environmental literacy	13.1	3	4.382	8.58	<0.001
	Abstract knowledge	41.4	3	13.799	21.63	<0.001
	Need for learning	26.6	3	8.86	14.03	<0.001
	Environmental citizenship	27.1	3	9.047	12.04	<0.001
	Need for action/Activism	32.1	3	10.712	26.78	<0.001
Residuals	Concrete knowledge	19245	696	27.651		
	Environmental awareness	363.9	696	0.523		
	Pro-environmental attitudes	183.6	696	0.264		
	Environmental (self-)education	782.9	696	1.125		
	Environmental literacy	355.5	696	0.511		
	Abstract knowledge	444	696	0.638		
	Need for learning	439.6	696	0.632		
	Environmental citizenship	522.9	696	0.751		
	Need for action/Activism	278.4	696	0.4		

**Table A3:** Means and standard deviations of all SMEC variables for each cluster.

	Cluster	M	SD
Concrete knowledge	1	16.6098	4.236
	2	16.3467	4.92
	3	17.2101	3.925
	4	10.9807	6.363
Environmental awareness	1	4.3008	0.715
	2	4.0503	0.682
	3	4.271	0.589
	4	3.6274	0.808
Pro-environmental attitudes	1	3.6618	0.605
	2	3.5745	0.527
	3	3.8291	0.519
	4	3.2355	0.45
Environmental (self-)education	1	3.039	1.201
	2	2.6724	1.034
	3	2.684	1.094
	4	2.9668	0.992
Environmental literacy	1	3.4324	0.746
	2	3.0685	0.63
	3	3.0466	0.791
	4	3.0927	0.724
Need for action/Activism	1	3.4946	0.63
	2	3.6884	0.506
	3	3.7535	0.427
	4	3.2407	0.782
Environmental citizenship	1	3.6938	0.921
	2	3.2915	0.772
	3	3.2689	0.918
	4	3.1248	0.885
Need for learning	1	3.639	0.897
	2	3.1899	0.779
	3	3.3227	0.815
	4	3.09	0.744
Abstract knowledge	1	3.9691	0.783
	2	3.5377	0.752
	3	3.5681	0.871
	4	3.2695	0.806

***Notes.*** Cluster are: 1—Positive; 2—Average; 3—Introverted, High in Neuroticism; 4—Negative.
